# National trends and territorial disparities in psychiatry residency fill rates in France: a longitudinal panel study, 2016–2026

**DOI:** 10.1016/j.hpopen.2026.100188

**Published:** 2026-10-10

**Authors:** Benoit Jambas, Marie Rouxel-Thomat, Arthur Girodeau, Julián Vadell Martínez, Francky Teddy Endomba

**Affiliations:** a Université Bourgogne Europe, Dijon, France; b Université Lyon 1, Villeurbanne, France; c CHU Dijon Bourgogne, Service de psychiatrie de l’adulte et de la personne âgée, Inserm, CTM UMR 1231, équipe PADYS, Dijon, France; d CIAD (Connaissance et Intelligence Artificielle Distribuées), UR 7533, Dijon, France

**Keywords:** Psychiatry, Health workforce, Residency training, Postgraduate medical education, Geographic variation, Physician distribution, France

## Abstract

**Background:**

Mental health needs are increasing across Europe, while psychiatric workforce planning in France is challenged by workforce ageing and territorial inequalities.

**Objective:**

To assess national trends in psychiatry residency fill rates in France and examine the territorial distribution of unfilled positions from 2016–2017 to 2025–2026.

**Methods:**

We conducted a nationwide retrospective longitudinal panel study including all 28 French residency subdivisions over 10 academic years. Opened and filled positions were extracted from decrees published in the French Official Journal. Fill rates were modelled using a beta-binomial generalised linear mixed model. Heterogeneity was quantified using the median odds ratio, and concentration using the Gini coefficient and top-share estimates.

**Results:**

The panel included 280 subdivision-year observations, corresponding to 5282 opened, 4719 filled, and 563 unfilled positions. Observed national fill rates declined from near-complete levels before 2019 to 82.49% in 2019–2020 and remained below pre-2019 levels thereafter. The model-based national fill rate decreased from 98.10% in the early period to 82.74% in the late period, an absolute change of −15.4 percentage points. Between-subdivision heterogeneity was substantial. Unfilled positions were territorially concentrated and the top 25% of subdivisions accounted for 61.3% of all unfilled positions.

**Conclusions:**

Psychiatry residency underfilling in France has become a sustained, territorially uneven workforce planning issue. In centralised rank-based allocation systems, workforce planning should monitor not only the number of training positions opened, but also their conversion into filled positions and the territorial accumulation of vacancies.

## Highlights

• Psychiatry residency underfilling threatens workforce renewal in France.
• Fill rates fell from 98.10% in 2016–19 to 82.74% in 2023–26.
• 563 positions remained unfilled, exceeding one average annual cohort.
• Seven subdivisions accounted for 61.3% of all unfilled positions.
• Workforce policy should prioritise territories with recurrent underfilling.

## Research in context box

**Table:** 

**What is already known about the topic?** *Recruitment to psychiatry is a persistent challenge internationally, and psychiatry has remained one of the least attractive specialities in France. Existing studies have mainly focused on speciality choice, rank-based attractiveness indicators, or determinants of applicants*' *preferences. However, little is known about whether nationally planned psychiatry residency positions are effectively filled, and how underfilling is distributed across territories.*
**What does this study add to the literature?** *Using a complete national panel of all 28 French residency subdivisions over 10 academic years, this study shows a substantial and sustained decline in psychiatry residency fill rates after 2019. It further demonstrates that underfilling was highly heterogeneous and territorially concentrated, with the top 25% of subdivisions accounting for 61.3% of all unfilled positions.*
**What are the policy implications?** *Workforce planning should monitor not only the number of psychiatry positions opened each year, but also whether these positions are filled and where deficits accumulate. Annual subdivision-level monitoring of opened, filled, and unfilled positions could identify territories with repeated underfilling and trigger targeted reviews of supervision capacity, training conditions, and regional workforce needs.*

## 1 Introduction

### 1.1 Background

Mental health needs are rising across Europe, increasing pressure on already overburdened systems and raising concerns about the capacity and distribution of the psychiatric workforce 1., 2., 3.. Across the World Health Organization (WHO) European Region, mental health conditions affect approximately one in six people, and one in three of those affected does not receive the treatment they need [4]. In France, national population-based surveillance data indicate that 12.5% of adults aged 18–85 years experienced a depressive episode in the preceding 12 months in 2021, with a marked upward trend since 2017, particularly among young adults [5]. These trends highlight the need for workforce planning capable of ensuring an adequate and geographically equitable psychiatric workforce.

Despite national regulation of medical training capacity, the French psychiatry workforce remains structurally fragile and territorially unequal. In 2025, 12533 psychiatrists were in regular practice nationally, yet this aggregate figure obscures two concerning features [6]. First, the workforce is ageing: the mean age of psychiatrists was 52 years in 2022, raising concerns about future replacement needs [7]. Second, the speciality is unevenly distributed across the territory. Departmental data from the French National Medical Council (Conseil National de l'Ordre des Médecins, CNOM) indicate persistent and widening territorial disparities in psychiatrist density [6]. These workforce constraints have concrete implications for access to care: in a 2023 national survey of public psychiatric facilities, 23% of psychiatrist positions were vacant, workforce shortages were the main reported reason for structural bed closures, and substantial delays in access to outpatient psychiatric care were reported [8]. Local psychiatrist density may also be associated with patterns of psychiatric service use; in a nationwide ecological study, each additional community-based private psychiatrist per 100,000 inhabitants was significantly associated with a 1.1% decrease in involuntary admission rates [9]. Together, these findings suggest that inequalities in psychiatrist availability may have implications for both access to mental health care and patterns of service use across outpatient and inpatient settings.

Residency training represents a key determinant of the physician workforce pipeline, through which health systems can influence the future supply and distribution of specialists 10., 11.. In the French centralised allocation system, the annual number of residency positions is determined nationally by speciality and subdivision, with each subdivision corresponding to a geographic training area in which residents undertake most of their rotations 12., 13.. The number and distribution of these openings are determined taking into account medical demography, territorial health needs, projected health-system requirements, and available training capacity, and therefore form part of national workforce planning [12]. Although specialists may practise anywhere in France and in any setting after residency, evidence from general practice suggests that residency allocation is associated with later settlement in the same area, lending empirical support to the workforce-planning rationale behind this policy [14]. The residency fill rate—the proportion of opened positions ultimately occupied—therefore captures both speciality recruitment and the extent to which training capacity planned according to national and territorial needs is filled, with potential implications for the distribution and availability of specialists.

Against a backdrop of workforce strain, recent concerns about declining fill rates have prompted the launch of the nationwide “Choose Psychiatry” (“Choisir psychiatrie”) campaign to encourage medical students to choose psychiatry as their speciality 8., 15., 16.. Psychiatry residency is a five-year programme defined nationally but implemented locally, with available rotations and training settings varying across subdivisions [13]. It comprises a two-year common foundation phase, after which residents choose between adult psychiatry and child and adolescent psychiatry, followed by a two-year advanced phase and a final one-year consolidation phase [13]. To identify previous studies relevant to this research gap, we conducted an exploratory literature search in PubMed. Each query combined “France” with English terms related to psychiatry, speciality choice, residency training, attractiveness, fill rates, and unfilled positions. The search was supplemented by Google Scholar using corresponding English and French search terms. This search identified previous French work on the determinants and perceptions shaping speciality choice, including psychiatry, as well as a recent nationwide study analysing speciality attractiveness using students' national rankings 16., 17., 18.. However, we found no peer-reviewed nationwide longitudinal study examining the extent, timing, and territorial distribution of psychiatry residency underfilling in France. We therefore aimed to assess national changes in psychiatry residency fill rates from 2016–2017 to 2025–2026, and to quantify between-subdivision heterogeneity in fill rates and the territorial concentration of unfilled training positions.

## 2 Methods

### 2.1 Study design, setting, and reporting guideline

We conducted a nationwide retrospective longitudinal observational panel study including all 28 French residency subdivisions over 10 academic years, from 2016–2017 to 2025–2026. The study is reported in accordance with the Strengthening the Reporting of Observational Studies in Epidemiology (STROBE) reporting guideline for observational studies [19].

### 2.2 Data sources, extraction, and quality control

Residency-position data were derived from decrees published in the French Official Journal (Journal Officiel de la République Française) and accessed through Légifrance, the official French government database for legislative and regulatory texts 20., 21.. The complete source inventory was documented in the study repository.

Filled-position data were automatically extracted using a custom script, then cleaned and harmonised through text normalisation and deterministic label mapping.

Opened-position data were manually digitised from scanned source tables by two investigators working independently; discrepancies were resolved by a third reviewer.

Scripted validation checks were applied to intermediate and final datasets to identify duplicate records, missing values, and internal inconsistencies.

### 2.3 Study population, statistical unit, and outcome definitions

All psychiatry subdivision-year observations in France from 2016–2017 to 2025–2026 were included. The statistical unit was the subdivision-year. The final analytic panel comprised all eligible statistical units, yielding 280 subdivision-year observations with no missing data; therefore, no sample size calculation was performed.

The primary outcome was the fill rate, defined as the number of filled positions divided by the total number of opened positions, including positions allocated through the *Contrat d'Engagement de Service Public* (CESP), a public-service commitment programme for eligible trainees. Unfilled positions were defined as opened positions minus filled positions. We prespecified an early period (2016–2017 to 2018–2019) and a late period (2023–2024 to 2025–2026).

### 2.4 Statistical analysis

We modelled the number of filled positions out of the total number of opened positions in each subdivision-year using a beta-binomial generalised linear mixed model (GLMM) with a logit link. Academic year was included as a fixed effect, and subdivision as a random intercept to account for repeated yearly observations and between-subdivision heterogeneity. A beta-binomial GLMM specification was used to account for extra-binomial variation.

Model-based national fill-rate estimates were obtained by weighting subdivision-year predictions by the number of opened positions. Prespecified early and late period estimates were obtained by combining academic-year-specific estimates within each period using the same weighting approach.

We reported the absolute difference, relative ratio, and odds ratio with 95% confidence intervals (CIs) derived from 2000 parametric bootstrap replicates. The contribution of academic year was assessed using a likelihood ratio test comparing models with and without the academic-year fixed effect. Statistical significance was defined at an alpha level of 0.05. As a sensitivity analysis, the model was refitted using a binomial GLMM specification.

Between-subdivision heterogeneity was quantified using the subdivision random-intercept variance and the median odds ratio. Territorial concentration of unfilled positions was described using a pooled Lorenz curve, the Gini coefficient, and the proportion of the cumulative national deficit contributed by the top 10% and top 25% of subdivisions ranked by cumulative unfilled positions. Subdivision-level descriptive indicators were reported in Supplementary Table A1.

All analyses were conducted in R using glmmTMB for mixed-effects modelling.

### 2.5 Contextual territorial analyses

For contextual territorial analyses, 2023 French National Institute of Statistics and Economic Studies (Institut national de la statistique et des études économiques, INSEE) population denominators and 2023 psychiatrist counts from the CNOM were aggregated to approximate residency subdivisions using departmental assignment rules. Psychiatrist density and residency-position indicators were expressed per 100,000 inhabitants. Late-period opened, filled, and unfilled positions were annualised over 2023–2026, while cumulative unfilled positions were calculated over 2016–2026. These descriptive indicators were not included in the primary models and are reported in Supplementary Table A2.

### 2.6 Ethics

This study used publicly available administrative decrees and did not involve individual-level or identifiable personal data; therefore, ethics approval and informed consent were not required.

## 3 Results

### 3.1 Analytic panel and national fill-rate trends

The panel included 280 subdivision-year observations (28 subdivisions over 10 academic years), corresponding to 5282 opened positions, 4719 filled, and 563 unfilled positions; no observations were excluded. Observed and model-based national fill rates showed a clear deterioration over time. Fill rates remained near-complete in the first three academic years (98.42%, 98.61%, and 96.42%), before dropping to 82.49% in 2019–2020. National fill rates subsequently remained below pre-2019 levels, reaching a low of 76.07% in 2024–2025, before partially recovering to 84.37% in 2025–2026 (Fig. 1).

**Fig. 1:**
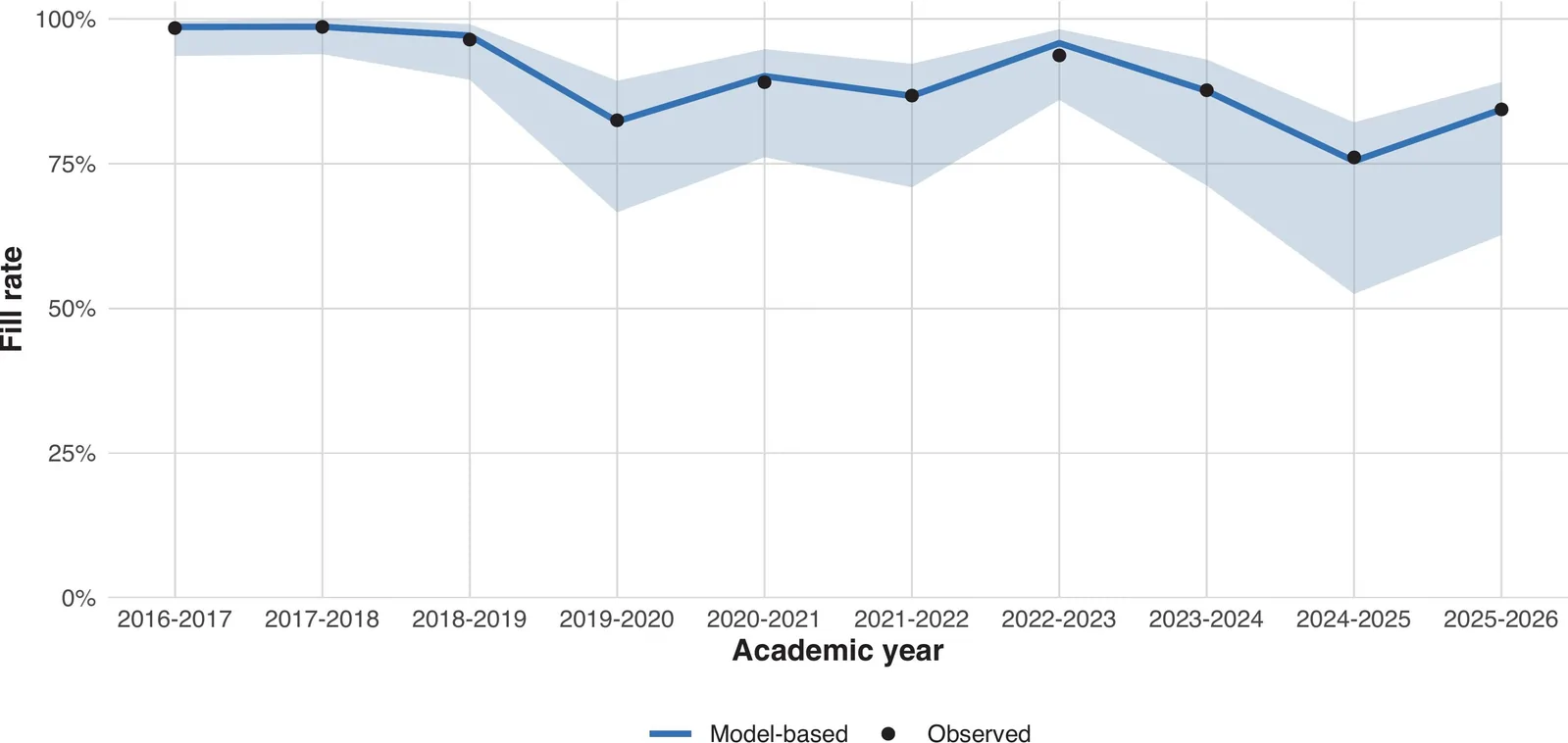
National psychiatry residency fill rates in France, 2016–2026. Observed and model-based fill rates by academic year. Shaded area: 95% CI.

### 3.2 Early-to-late change in fill rates

Comparing the early and late periods, the model-based national fill rate decreased from 98.10% to 82.74%, corresponding to an absolute change of −15.4 percentage points (95% CI, −31.0 to −11.2) and a relative ratio of 0.843 (95% CI, 0.674 to 0.886). The corresponding odds ratio is reported in Table 1. There was strong evidence that including academic year as a fixed effect improved model fit overall, compared with a model without the academic-year effect (likelihood ratio test, *p* < 0.001).

**Table 1:** Model-based estimates of psychiatry residency fill rates and between-subdivision heterogeneity.

Domain	Metric	Estimate	95% CI
Primary analysis	Model-based national fill rate, EARLY period (2016–2017 to 2018–2019)	98.10%	[93.26; 99.16]
Primary analysis	Model-based national fill rate, LATE period (2023–2024 to 2025–2026)	82.74%	[63.87; 87.42]
Primary analysis	Absolute change in fill rate, LATE minus EARLY (percentage points)	−15.4	[−31.0; −11.2]
Primary analysis	Relative ratio, LATE vs EARLY	0.843	[0.674; 0.886]
Primary analysis	Odds ratio, LATE vs EARLY	0.093	[0.039; 0.188]
Subdivision heterogeneity	Between-subdivision variance (random intercept, logit scale)	2.933	[1.335; 5.389]
Subdivision heterogeneity	Median odds ratio (MOR)	5.122	[3.010; 9.156]
Sensitivity analysis	Relative ratio, LATE vs EARLY (binomial)	0.848	[0.668; 0.892]

Notes: CI, confidence interval; EARLY, 2016–2017 to 2018–2019; LATE, 2023–2024 to 2025–2026; MOR, median odds ratio; pp., percentage points.

### 3.3 Between-subdivision heterogeneity

There was strong evidence of between-subdivision heterogeneity. The estimated random-intercept variance was 2.933 (95% CI, 1.335 to 5.389). The median odds ratio was 5.122 (95% CI, 3.010 to 9.156), meaning that two randomly chosen subdivisions differed by about fivefold in the median odds of filling a psychiatry residency position. The heatmap of observed fill rates made this heterogeneity visually apparent, with persistent between-subdivision differences across academic years (Fig. 2).

**Fig. 2:**
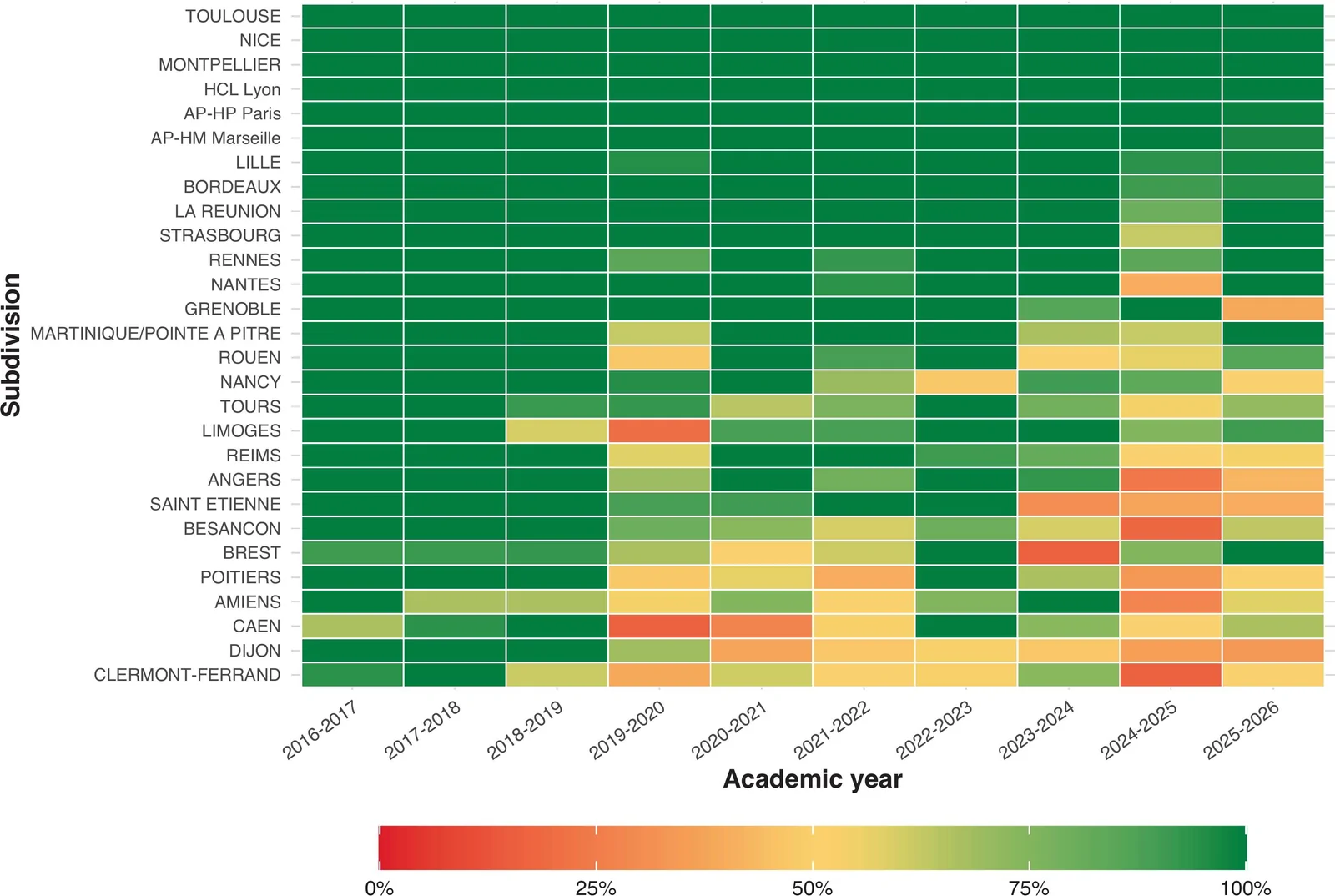
Heatmap of observed psychiatry residency fill rates by subdivision and academic year. Warmer colours indicate lower fill rates. Subdivisions are ordered by cumulative unfilled positions.

### 3.4 Territorial concentration and contextual indicators

Cumulative unfilled positions over 2016–2026 were markedly concentrated across subdivisions. The Lorenz curve showed a substantial deviation from the line of equality, with a Gini coefficient of 0.542. The top 10% and top 25% of subdivisions, corresponding to the 3 and 7 subdivisions with the largest cumulative deficits, accounted for 32.1% and 61.3%, respectively, of the 563 unfilled positions recorded over 2016–2026 (Fig. 3).

**Fig. 3:**
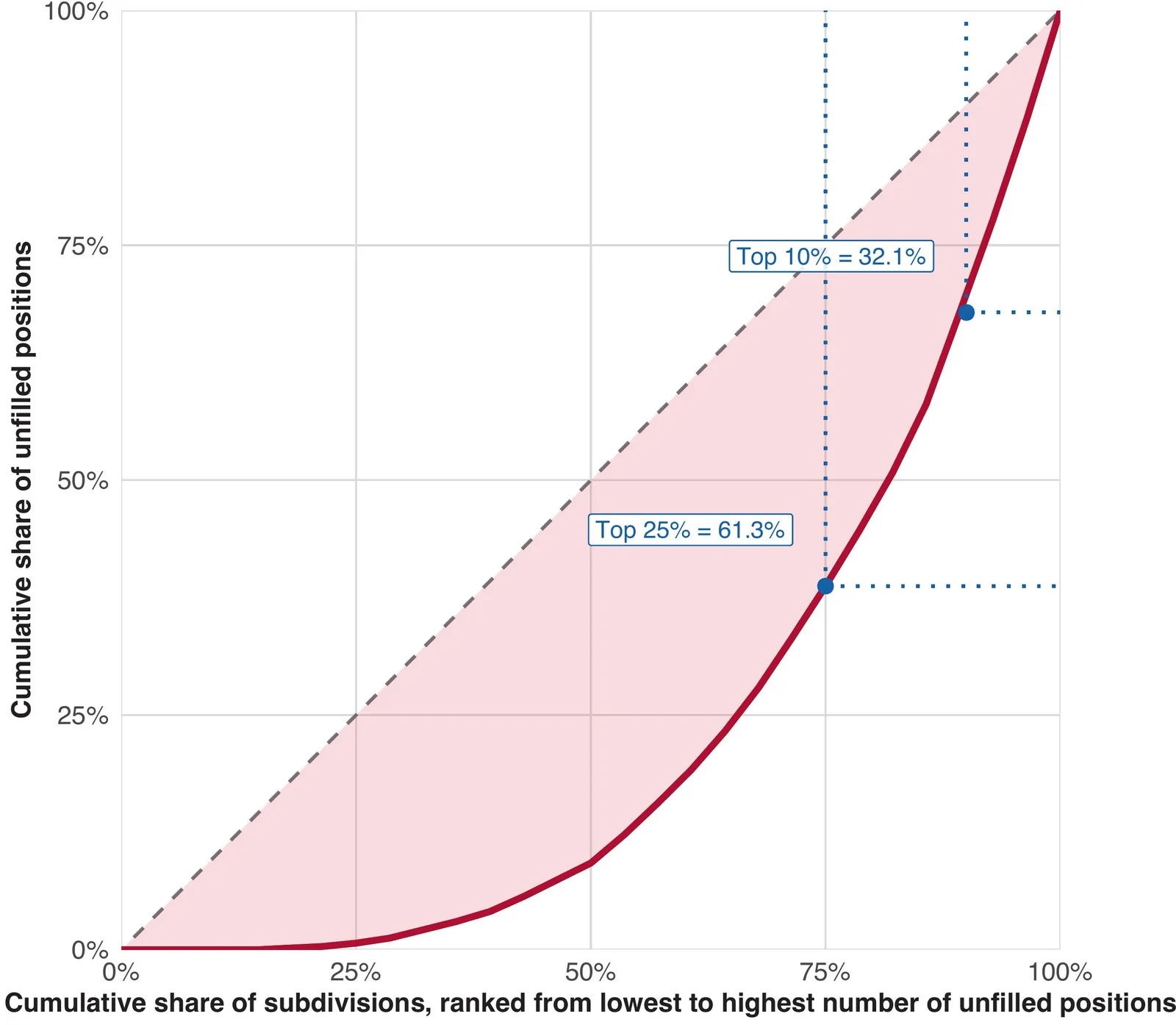
Territorial concentration of unfilled psychiatry residency positions, 2016–2026. Lorenz curve of cumulative unfilled positions across subdivisions. Gini = 0.542; top 10% and 25% accounted for 32.1% and 61.3% of unfilled positions.

Four subdivisions accounted for 236 of the 563 unfilled positions (41.9%): Clermont-Ferrand, Dijon, Poitiers, and Caen. Their population-standardised deficits ranged from 3.05 to 4.74 per 100,000 inhabitants, alongside psychiatrist density of 12.83 to 17.51 per 100,000. By contrast, Hospices Civils de Lyon (HCL Lyon), Montpellier, Nice, and Toulouse recorded no unfilled positions; Assistance Publique–Hôpitaux de Paris (AP-HP Paris), Assistance Publique–Hôpitaux de Marseille (AP-HM Marseille), HCL Lyon, and Nice had no or near-zero deficits with higher psychiatrist density, ranging from 21.11 to 25.83 per 100,000 (Supplementary Tables A1–A2).

The largest early-to-late declines in fill rates were observed in Saint-Étienne (−64.29 percentage points), Dijon (−60.87), Besançon (−51.35), and Poitiers (−48.98; Supplementary Table A1). Results were similar in the binomial sensitivity analysis, with a relative ratio of 0.848 (95% CI, 0.668 to 0.892), consistent with the primary analysis.

## 4 Discussion

### 4.1 Principal findings

This nationwide longitudinal panel study examined whether psychiatry residency positions opened in France were effectively filled over 2016–2026 and how unfilled positions were distributed across subdivisions. We found a substantial and sustained decline in psychiatry residency fill rates in France over 2016–2026, with the model-based national fill rate declining from 98.10% in the early period to 82.74% in the late period. This decline was highly uneven: underfilling varied markedly across subdivisions and was disproportionately concentrated in a small number of territories. In practical terms, the 563 positions that remained unfilled over the decade exceeded one average annual national cohort of opened psychiatry positions. Taken together, these findings indicate that psychiatry underfilling has become a major workforce planning issue in France.

### 4.2 Interpretation in the context of the French allocation system

Recruitment to psychiatry is widely recognised as a global challenge, yet recruitment difficulties may reflect different mechanisms depending on the allocation system through which candidates enter speciality training 22., 23., 24.. European systems differ in how residency positions are planned, candidates selected and ranked, preferences expressed, and speciality and location allocated 22., 24., 25., 26.. Comparative studies have begun to map these differences, but they have not yet established how different allocation models affect the translation of workforce planning into realised recruitment, the territorial distribution of vacancies, and the effectiveness of policy responses 24., 25., 26.. Portugal and Spain are close comparators to France because they allocate candidates, through national examination-based rankings, to a finite set of speciality–location positions 24., 27.. Both have reported territorially uneven unfilled residency positions, mainly in general practice rather than psychiatry 28., 29., 30., 31.. These cases illustrate a key mechanism of centralised rank-based allocation systems: when a speciality is less attractive than competing options, candidates may leave some speciality–subdivision positions unfilled, allowing candidates' choice to reshape the planned distribution. Observed choices therefore do not directly reflect candidates' true preferences; they are constrained by rank and by the positions still available when each candidate's turn arrives [23]. We consequently interpret our findings as the interaction between allocation, through which policy defines the number and territorial distribution of openings, and attractiveness, through which candidates choose among the remaining options.

These model-specific features make psychiatry recruitment difficulties less directly comparable across allocation systems and limit the transferability of policy responses. In the United Kingdom, which inspired the French #ChoisirPsychiatrie campaign, preferences are expressed mainly at the application stage: applicants decide whether and when to apply, may apply to several specialities, and rank geographical preferences 32., 33., 34., 35., 36., 37.. Speciality attractiveness therefore plays a more direct role within this application-based recruitment system [38].

Against this background, our study extends previous work by providing longitudinal evidence of three distinct findings: the emergence of territorially uneven recruitment difficulties within a centralised rank-based allocation system, their occurrence in psychiatry rather than general practice, and their abrupt onset.

### 4.3 Potential drivers of the decline in psychiatry fill rates

Within this interpretive framework, the temporal pattern is particularly informative. Psychiatry fill rates dropped sharply in 2019, corresponding to the 2019–2020 academic year, from 96.42% in 2018–2019 to 82.49%, and remained below pre-2019 levels thereafter. This abrupt decline suggests that the factors influencing fill rates, or their relative importance, changed around 2019.

At the national level, one possible explanation is an increase in overall position-candidate mismatch: when the number of opened positions exceeds the number of candidates, some positions necessarily remain unfilled. However, the available data do not support overall mismatch as the primary explanation for the deterioration observed from 2019 onward. Psychiatry was fully filled in 2011 despite a high mismatch rate of 9% [39]. Following the 2011 reform limiting opportunities for deferral, mismatch levels fell and remained broadly stable from 2012 onward [27]. Importantly, the abrupt decline in psychiatry fill rates occurred in a lower-mismatch context, with a mismatch rate of 2.3% in 2019 compared with 4.2% in 2018 [27]. At the same time, the available speciality-level reports suggest that the 2019 breakpoint coincided with a redistribution of unfilled positions across lower-attractiveness specialities 18., 27.. Between 2018 and 2019, unfilled positions fell sharply in general practice (183 to 13), occupational medicine (48 to 20), emergency medicine (21 to 7), and clinical biology (20 to 8), while rising markedly in psychiatry (19 to 93) and more modestly in public health (21 to 31) [27]. This pattern suggests a possible shift from a distribution of vacancies resembling other rank-based systems, where unfilled positions have mainly concerned general practice, toward a French configuration in which vacancies became concentrated in psychiatry 28., 30., 31.. Because later comparable data were unavailable, this pattern cannot establish sustained redistribution. However, recent longitudinal evidence relying on rank-based indicators of speciality attractiveness may have missed this shift [18]. Combining rank-based indicators with unfilled-position data could therefore provide a more comprehensive assessment of attractiveness over time, particularly when vacancies are redistributed among less attractive specialities.

Psychiatry is affected by structural and longstanding determinants of lower attractiveness, documented both internationally and in France, including stigma, low prestige, and perceptions of the speciality as less scientific 17., 22.. However, these factors cannot be the sole explanation of the abrupt decline observed in 2019. More modifiable determinants, including clinical exposure, supervision, training quality, working conditions, and work-life balance, offer more concrete intervention targets 17., 22., 40.. Broader institutional problems, such as understaffing, shortages of trainers, and concerns about quality of care, may reduce psychiatry's attractiveness by weakening the training environments through which applicants develop positive professional identification [22]. In France, the 2019 breakpoint also coincided with heightened media and institutional attention to structural difficulties in public psychiatry, including the Le Rouvray hunger strike, professional mobilisation, and a parliamentary report on understaffing and deteriorating working conditions 41., 42., 43.. These events cannot establish causality, but they provide contextual evidence for why pre-existing structural difficulties may have become more salient to applicants around the time of the observed breakpoint. COVID-19 cannot explain the initial breakpoint, which preceded the pandemic, but may have exacerbated underfilling thereafter 44., 45., 46..

### 4.4 Territorial concentration, policy implications, and potential responses

Our findings raise important questions about the policy framework used to allocate residency positions. Although the national volume of opened psychiatry positions remained broadly stable between the early and late periods (+4.0%), this stability masked substantial territorial redistribution. Some subdivisions with worsening fill rates reduced their openings, such as Clermont-Ferrand (57 to 41; −16; 17.51 psychiatrists per 100,000 inhabitants) and Poitiers (61 to 49; −12; 12.83 per 100,000), whereas subdivisions with consistently high fill rates expanded their openings while continuing to fill them, including AP-HP Paris (281 to 345; +64; 25.83 per 100,000) and HCL Lyon (70 to 83; +13; 21.43 per 100,000; Supplementary Tables A1–A2) [6]. Although psychiatrist density is an imperfect proxy for workforce need, these patterns do not suggest a straightforward compensatory allocation policy toward lower-density or worsening-fill subdivisions (Supplementary Table A2). This does not imply that allocation decisions were inappropriate, since they may also reflect local training capacity, supervision, or service constraints. Rather, they echo analyses from France and other centralised systems showing that state planning instruments have struggled to correct territorial inequalities in physician distribution 47., 48., 49.. Underfilling amplifies this problem: training capacity planned according to national and territorial needs does not necessarily translate into filled positions, leaving candidates' preferences to shape the effective distribution of filled positions [50]. Although residency allocation is intended to support local workforce renewal and training location has been associated with later settlement in general practice, further research should determine whether similar patterns occur in psychiatry and across practice settings 12., 14..

Beyond the national trend, our findings reveal substantial and persistent between-subdivision heterogeneity, quantified by a median odds ratio of 5.122. Because psychiatry training is implemented locally within subdivisions, differences in local training environments may contribute to this heterogeneity and represent a modifiable policy target [13]. Previous work indicates that local attractiveness is shaped by metropolitan size, quality of life in the host region, local medical density, the training environment, and the ability to attract residents from other regions 17., 51., 52.. Persistent underfilling could plausibly generate a negative feedback loop by affecting several modifiable dimensions of attractiveness: a smaller resident workforce may increase workload and on-call burden, strain working conditions, reduce supervision capacity, and weaken perceived training quality 17., 51., 53.. In a centralised rank-based allocation system, these mechanisms may support targeted interventions addressing the attractiveness of specific speciality–subdivision positions 23., 54..

### 4.5 Generalisability, strengths, and limitations

Our findings are most applicable to centralised rank-based systems allocating candidates to finite speciality–location positions, with more cautious transferability to allocation models where selection, preference expression, or speciality–location allocation differ.

Several limitations should be considered. First, because the datasets were reconstructed from administrative sources not designed for research, residual extraction, transcription, or harmonisation errors cannot be excluded, despite double independent digitisation, adjudication, and scripted quality checks. Second, the descriptive observational design precludes causal inference and assessment of policy decision-making.

This study's main strengths are its exhaustive national coverage, reproducible administrative data, and combined use of mixed-effects modelling and territorial concentration analyses. Inference was strengthened by accounting for repeated subdivision-year observations, quantifying uncertainty, and confirming robustness in sensitivity analyses.

## 5 Conclusions

In centralised rank-based allocation systems, opened positions define the planned distribution, but they do not ensure workforce renewal unless they are converted into filled positions. Persistent underfilling therefore represents both unrealised recruitment and a potential source of territorial inequalities in workforce renewal, with possible implications for access to psychiatric care. This study suggests that the decline in psychiatry residency fill rates should be understood not only as a recruitment issue, but also as a concern for workforce planning with potential implications for territorial equity.

Policy responses should move beyond the annual number of psychiatry positions opened and consider whether these positions are likely to be filled, aligned with regional mental health needs, and supported by sufficient local training capacity and supervision. Future research should examine how applicants choose specialities and subdivisions, identify modifiable features of local training environments, and assess whether repeated underfilling affects local workforce renewal and access to psychiatric care. Integrating these dimensions could support more anticipatory and equity-oriented planning for the psychiatric workforce.

## Declaration of generative AI and AI-assisted technologies in the manuscript preparation process

During the preparation of this work, the authors used ChatGPT to improve readability. After using this tool, the authors reviewed and edited the content as needed and take full responsibility for the content of the submitted manuscript.

## Author confirmation

● All authors meet authorship criteria.
● All authors approved the submitted version.
● All authors agree to be accountable for all aspects of the work.
● The manuscript is not under consideration elsewhere.

## CRediT authorship contribution statement

**Benoit Jambas:** Writing – review & editing, Writing – original draft, Validation, Software, Project administration, Methodology, Investigation, Formal analysis, Data curation, Conceptualization. **Marie Rouxel-Thomat:** Writing – review & editing, Writing – original draft, Data curation. **Arthur Girodeau:** Writing – review & editing, Writing – original draft. **Julián Vadell Martínez:** Writing – review & editing, Supervision, Methodology, Data curation. **Francky Teddy Endomba:** Writing – review & editing, Supervision, Methodology.

## Funding statement

This research did not receive any specific grant from funding agencies in the public, commercial, or not-for-profit sectors.

## Declaration of competing interest

The authors declare that they have no known competing financial interests or personal relationships that could have appeared to influence the work reported in this paper.

## Data Availability

The analytic dataset was reconstructed from publicly available French Official Journal decrees. The analysis code and data supporting the findings of this study are available for peer review in an anonymized private repository: https://doi.org/10.17605/OSF.IO/UNSCA

## Appendix A Supplementary data

Supplementary materialSubdivision-level psychiatry residency fill rates, unfilled positions, and contextual workforce indicators in France, 2016-2026.
