# Supplementary material for: National trends and territorial disparities in psychiatry residency fill rates in France: a longitudinal panel study, 2016–2026
Source: Health Policy Open. 2026 Oct 10;11:100188. doi: 10.1016/j.hpopen.2026.100188 (PMC13694877; doi:10.1016/j.hpopen.2026.100188)
Supplement: Supplementary file 1 [file mmc1.docx]

Appendix / e-component

# Appendix A. Supplementary results

# Table A1. Subdivision-level opened positions, fill rates and unfilled positions

| **Subdivision** | **Opened positions, early period** | **Fill rate, early period (%)** | **Opened positions, late period** | **Fill rate, late period (%)** | **Absolute change, late vs early (pp)** | **Relative change, late vs early** | **Cumulative unfilled positions, 2016–2026** | **Share of national unfilled positions (%)** |
| --- | --- | --- | --- | --- | --- | --- | --- | --- |
| **ALL** | 1541 | 97.79 | 1604 | 82.98 | -14.81 | 0.849 | 563 | 100 |
| **CLERMONT-FERRAND** | 57 | 84.21 | 41 | 48.78 | -35.43 | 0.579 | 65 | 11.55 |
| **DIJON** | 47 | 100 | 46 | 39.13 | -60.87 | 0.391 | 60 | 10.66 |
| **POITIERS** | 61 | 100 | 49 | 51.02 | -48.98 | 0.51 | 56 | 9.95 |
| **CAEN** | 53 | 86.79 | 41 | 63.41 | -23.38 | 0.731 | 55 | 9.77 |
| **AMIENS** | 45 | 77.78 | 35 | 62.86 | -14.92 | 0.808 | 41 | 7.28 |
| **BESANCON** | 43 | 100 | 37 | 48.65 | -51.35 | 0.486 | 35 | 6.22 |
| **NANCY** | 52 | 100 | 58 | 74.14 | -25.86 | 0.741 | 33 | 5.86 |
| **BREST** | 36 | 91.67 | 28 | 57.14 | -34.52 | 0.623 | 31 | 5.51 |
| **ROUEN** | 59 | 100 | 46 | 63.04 | -36.96 | 0.63 | 30 | 5.33 |
| **ANGERS** | 36 | 100 | 41 | 53.66 | -46.34 | 0.537 | 26 | 4.62 |
| **TOURS** | 34 | 97.06 | 41 | 68.29 | -28.77 | 0.704 | 23 | 4.09 |
| **SAINT ETIENNE** | 27 | 100 | 28 | 35.71 | -64.29 | 0.357 | 20 | 3.55 |
| **REIMS** | 28 | 100 | 34 | 61.76 | -38.24 | 0.618 | 19 | 3.37 |
| **LIMOGES** | 29 | 86.21 | 28 | 89.29 | 3.08 | 1.036 | 17 | 3.02 |
| **GRENOBLE** | 34 | 100 | 38 | 73.68 | -26.32 | 0.737 | 10 | 1.78 |
| **NANTES** | 42 | 100 | 47 | 80.85 | -19.15 | 0.809 | 10 | 1.78 |
| **MARTINIQUE/POINTE A PITRE** | 21 | 100 | 27 | 77.78 | -22.22 | 0.778 | 9 | 1.6 |
| **STRASBOURG** | 42 | 100 | 51 | 88.24 | -11.76 | 0.882 | 6 | 1.07 |
| **LILLE** | 117 | 100 | 111 | 97.3 | -2.7 | 0.973 | 5 | 0.89 |
| **RENNES** | 34 | 100 | 39 | 94.87 | -5.13 | 0.949 | 5 | 0.89 |
| **BORDEAUX** | 66 | 100 | 66 | 95.45 | -4.55 | 0.955 | 3 | 0.53 |
| **AP-HP Paris** | 281 | 100 | 345 | 99.42 | -0.58 | 0.994 | 2 | 0.36 |
| **AP-HM Marseille** | 73 | 100 | 76 | 98.68 | -1.32 | 0.987 | 1 | 0.18 |
| **LA REUNION** | 12 | 100 | 17 | 94.12 | -5.88 | 0.941 | 1 | 0.18 |
| **HCL Lyon** | 70 | 100 | 83 | 100 | 0 | 1 | 0 | 0 |
| **MONTPELLIER** | 44 | 100 | 46 | 100 | 0 | 1 | 0 | 0 |
| **NICE** | 30 | 100 | 35 | 100 | 0 | 1 | 0 | 0 |
| **TOULOUSE** | 68 | 100 | 70 | 100 | 0 | 1 | 0 | 0 |

Notes: EARLY, 2016-2017 to 2018-2019; LATE, 2023-2024 to 2025-2026.

# Table A2. Contextual subdivision-level indicators of psychiatrist density and psychiatry residency training capacity, 2023

| **Subdivision** | **Population, 2023** | **Psychiatrists, 2023** | **Psychiatrist density per 100,000 inhabitants, 2023** | **Late-period opened positions per 100,000 inhabitants per year** | **Late-period filled positions per 100,000 inhabitants per year** | **Late-period unfilled positions per 100,000 inhabitants per year** | **Cumulative unfilled positions per 100,000 inhabitants, 2016–2026** |
| --- | --- | --- | --- | --- | --- | --- | --- |
| **CLERMONT-FERRAND** | 1370601 | 240 | 17.51 | 0.997 | 0.486 | 0.511 | 4.742 |
| **CAEN** | 1482164 | 197 | 13.29 | 0.922 | 0.585 | 0.337 | 3.711 |
| **DIJON** | 1624094 | 229 | 14.1 | 0.944 | 0.369 | 0.575 | 3.694 |
| **POITIERS** | 1839088 | 236 | 12.83 | 0.888 | 0.453 | 0.435 | 3.045 |
| **BESANCON** | 1178576 | 211 | 17.9 | 1.046 | 0.509 | 0.537 | 2.97 |
| **SAINT ETIENNE** | 774133 | 117 | 15.11 | 1.206 | 0.431 | 0.775 | 2.584 |
| **LIMOGES** | 729520 | 123 | 16.86 | 1.279 | 1.142 | 0.137 | 2.33 |
| **AMIENS** | 1918654 | 236 | 12.3 | 0.608 | 0.382 | 0.226 | 2.137 |
| **BREST*** | 1545314 | 252 | 16.31 | 0.604 | 0.345 | 0.259 | 2.006 |
| **ROUEN** | 1863678 | 279 | 14.97 | 0.823 | 0.519 | 0.304 | 1.61 |
| **ANGERS** | 1705977 | 229 | 13.42 | 0.801 | 0.43 | 0.371 | 1.524 |
| **REIMS** | 1307747 | 171 | 13.08 | 0.867 | 0.535 | 0.331 | 1.453 |
| **NANCY** | 2321083 | 315 | 13.57 | 0.833 | 0.618 | 0.215 | 1.422 |
| **TOURS** | 2587031 | 309 | 11.94 | 0.528 | 0.361 | 0.168 | 0.889 |
| **MARTINIQUE/POINTE A PITRE** | 1038786 | 139 | 13.38 | 0.866 | 0.674 | 0.193 | 0.866 |
| **NANTES** | 2201179 | 362 | 16.45 | 0.712 | 0.575 | 0.136 | 0.454 |
| **GRENOBLE*** | 2608374 | 420 | 16.1 | 0.486 | 0.358 | 0.128 | 0.383 |
| **STRASBOURG** | 1934548 | 355 | 18.35 | 0.879 | 0.775 | 0.103 | 0.31 |
| **RENNES*** | 1904056 | 313 | 16.44 | 0.683 | 0.648 | 0.035 | 0.263 |
| **LILLE*** | 4073540 | 633 | 15.54 | 0.908 | 0.884 | 0.025 | 0.123 |
| **BORDEAUX** | 3581843 | 732 | 20.44 | 0.614 | 0.586 | 0.028 | 0.084 |
| **LA REUNION** | 1200242 | 179 | 14.91 | 0.472 | 0.444 | 0.028 | 0.083 |
| **AP-HM Marseille*** | 4258848 | 899 | 21.11 | 0.595 | 0.587 | 0.008 | 0.023 |
| **AP-HP Paris** | 12463067 | 3219 | 25.83 | 0.923 | 0.917 | 0.005 | 0.016 |
| **HCL Lyon*** | 3452449 | 740 | 21.43 | 0.801 | 0.801 | 0 | 0 |
| **MONTPELLIER*** | 2954218 | 512 | 17.33 | 0.519 | 0.519 | 0 | 0 |
| **NICE*** | 1315598 | 316 | 24.02 | 0.887 | 0.887 | 0 | 0 |
| **TOULOUSE*** | 3170435 | 570 | 17.98 | 0.736 | 0.736 | 0 | 0 |

**Note:** Asterisks identify subdivisions whose population denominators involved at least one department with infra-departmental exceptions or overlapping assignment in the legal residency-subdivision definition from (https://www.legifrance.gouv.fr/loda/id/JORFTEXT000035589599). This concerned **9/28 subdivisions**; the remaining **19/28 subdivisions** were based on direct department-to-subdivision aggregation. In affected cases, each department was assigned once using a predefined main-part or department-specific rule: 07→HCL Lyon; 11→Montpellier; 12→Toulouse; 22→Brest; 2A→AP-HM Marseille; 2B→Nice; 38→Grenoble; 56→Rennes; 62→Lille; 74→Grenoble; 83→AP-HM Marseille. Var (83) was assigned to AP-HM Marseille, and the Giens/Hyères hospital exception was not assigned a separate population. These indicators are descriptive contextual approximations and should not be interpreted as exact legal subdivision denominators; they were not included in the primary models
